# The value of systemic immune inflammation index, white blood cell to platelet ratio, and homocysteine in predicting the instability of small saccular intracranial aneurysms

**DOI:** 10.1038/s41598-024-74870-y

**Published:** 2024-10-16

**Authors:** Wanwan Zhang, Chao Xiang, Boliang Liu, Fandi Hou, Zhanqiang Zheng, Zhongcan Chen, Lina Suo, Guang Feng, Jianjun Gu

**Affiliations:** 1 Department of Neurosurgery, Henan Provincial People’s Hospital Affiliated to Henan University, Zhengzhou, Henan People’s Republic of China; 2 Department of Clinical Medicine, Henan University, Kaifeng, Henan People’s Republic of China; 3 Department of Neurosurgery, Henan Provincial People’s Hospital Affiliated to Zhengzhou University, Zhengzhou, Henan People’s Republic of China

**Keywords:** Small saccular intracranial aneurysms, Systemic immune-inflammatory index, White blood cell and platelet ratio, Homocysteine, Inflammatory marker, Rupture risk, Neuroscience, Biomarkers, Medical research, Neurology

## Abstract

Inflammation has a destructive effect on the homeostasis of the vascular wall, which is involved in the formation, growth, and rupture of human intracranial aneurysms (IAs) disease progression. However, inflammation-related markers have not been well studied in the risk stratification of unruptured IAs. The purpose of this study was to investigate the predictive value of serum inflammatory markers in the unstable progression of small saccular intracranial aneurysms (SIAs). This study retrospectively included 275 patients with small SIAs (aneurysm diameter less than or equal to 7 mm), to compare the level difference of serum inflammatory complex marker systemic immune-inflammatory index (SII), white blood cell to platelet ratio (WPR), and homocysteine (Hcy) in patients with stable (asymptomatic unruptured) and unstable (symptomatic unruptured, ruptured) small SIAs. 187 patients (68%) had aneurysm-related compression symptoms and rupture outcomes. In the multivariate logistic regression after adjusting for baseline differences, SII, WPR, and Hcy were independent risk factors for the instability of small SIAs, the prediction model combined with other risk factors (previous stroke history, aneurysm irregularity) showed good predictive ability for the instability of small SIAs, with an area under the curve of 0.905. In addition, correlation analysis showed that SII, WPR, and Hcy also had significant differences in patients with symptomatic unruptured and ruptured small SIAs, and higher inflammation levels often promoted the disease progression of small SIAs. Higher levels of SII, WPR and Hcy can be used as independent predictors of instability of small SIAs. As an economical and convenient biomarker, it is crucial for clinical treatment strategies of stable small SIAs.

## Introduction

Intracranial aneurysms (IAs) are localized and pathological expansion of the intracranial artery wall due to risk factors such as damage to the internal elastic layer and the destruction of media^1^. Previous studies have reported that the prevalence of IAs in the general population is about 2-3%^2^. Even if the prevalence is not high, IAs rupture is the main cause for 85% of non-traumatic subarachnoid hemorrhage^3^, aneurysmal subarachnoid hemorrhage (aSAH) often brings disastrous consequences to patients, leading to serious disability and mortality.

According to the morphological characteristics, IAs can be roughly divided into saccular and non-saccular aneurysms, of which the former is the most common, accounting for about 90% of all IAs^4^. Saccular intracranial aneurysms (SIAs) refers to the abnormal round bulge from the side wall of the artery, which mostly occur at the junctions or transition points of the intracranial vascular system, such as bifurcations and terminals, and the prevalence of SIAs in the total population is about 1–2%^4,5^. Combined with clinical practice, most SIAs are stable and have a low risk of rupture, especially some small aneurysms. A previous international retrospective study found that the risk of rupture of IAs with diameter ≥ 10 mm was 11.6–59 times that of IAs with diameter < 10 mm, which confirmed that the risk of rupture of IAs was proportional to its size^6^. However, paradoxically, most ruptured aneurysms were found to be small aneurysms in clinical practice, far exceeding the proportion of large aneurysms. In one large retrospective study, 71.8% of ruptured aneurysms were found to be less than 7 mm in diameter, with an average diameter of 6.28 mm^7^. Another retrospective study of aSAH found that 75% of patients were caused by IA (≤ 7 mm ), and the median ruptured IA size was 5.3 mm^8^. In addition, a number of international prospective studies, including International Study of Unruptured Intracranial Aneurysms (ISUIA) and Unruptured Cerebral Aneurysms Study (UCAS), have used 7 mm as the critical point of small IAs to assess their growth and rupture risk^9,10^. It is worth noting that, even if some aneurysms have the same size, those with significant clinical symptoms and evidence of growth during follow-up have a higher risk of rupture, these and ruptured aneurysms are often clinically classified as unstable aneurysm types^11,12^. Considering that the rupture of IAs may cause serious damage to the central nervous system, it is particularly important to accurately identify unstable and easily ruptured small SIAs in the early stage and give timely treatment and intervention measures.

At present, although medical technology has made great progress in clinical diagnosis and treatment, the mechanism of formation and growth of intracranial aneurysms has not been fully understood. A large number of studies have shown that hemodynamic changes, inflammatory pathway activation and atherosclerosis may be involved in the formation and unstable progression of intracranial aneurysms^13–15^. Studies have pointed out that endothelial cells at the bifurcation of large blood vessels are prone to dysfunction under the influence of hemodynamics, which in turn leads to inflammatory reactions of various cytokines, inflammatory mediators, macrophages, neutrophils, etc^16^. The inflammatory reaction leads to the destruction of the internal elastic layer and eventually forms an aneurysm, further inflammatory reaction and vascular wall degeneration eventually lead to IAs rupture^17^. Based on this, our study focuses on the role of serum inflammatory markers in the development of small SIAs disease from the perspective of inflammation.

As a common clinical indicator of the systemic inflammatory response, serum inflammatory markers such as white blood cells, neutrophils, C-reactive protein, procalcitonin, interleukin, and serum amyloid protein have been widely studied in many diseases to help predict the disease process and prognosis and optimize clinical treatment decisions^18–21^. Influenced by the key role of inflammatory factors in the process of other diseases, some scholars have designed a large number of clinical and animal experiments following the theory of intracranial aneurysm progression mechanism in recent years, and the results have confirmed that inflammation is involved in the occurrence and development of the disease and is considered to be a key factor in the growth and rupture of aneurysms. It has been found that high levels of IL-1, cholesterol, ratio of IL-1.ra to Il-1β, TNF-α and low ratio of platelets to neutrophils as serum biomarkers have good potential value in identifying unstable aneurysms (symptomatic, growing or ruptured). Although some serum biomarkers have been reported to be useful for monitoring IAs disease progression, there has been little research in the most common type of small SIAs in clinical practice^22–25^. The purpose of this study is to explore the predictive significance of SII, WPR, and Hcy in the disease progression of patients with confirmed small SIAs. By finding reliable serum inflammatory markers, we can better assess the risk of secondary instability and rupture bleeding in patients with stable small SIAs to make clinical decisions that are in the best interests of patients from a safe and economic perspective.

## Materials and methods

### Research object

Our retrospective cohort study collected patients with small SIAs (diameter ≤ 7 mm) diagnosed and treated in Henan Provincial People’s Hospital from January 2022 to June 2023, including 88 patients with stable asymptomatic aneurysms, 88 patients with unstable symptomatic unruptured aneurysms, and 99 patients with ruptured aneurysms. The criteria for unstable SIAs were defined as aneurysms with adjacent structural compression accompanied by acute or chronic neurological symptoms, aneurysms growing at any follow-up (compared to baseline), and IAs that ruptured within 24 hours. Other asymptomatic aneurysms found accidentally were classified as stable^11,12^. All patients underwent computed tomography angiography (CTA)/magnetic resonance angiography (MRA)/digital subtraction angiography (DSA) to detect IAs, and finally further diagnosed by DSA. The aneurysm diameter was manually measured from the best working angle selected from the three-dimensional DSA image. The following criteria were excluded: (1) Aneurysm patients younger than 18 years. (2) Patients with aneurysm diameter greater than 7 mm. (3) Unstable ruptured aneurysms in patients with onset ≥ 24 h. (4) Patients with cerebrovascular malformation, moyamoya disease, arteriovenous fistula, brain tumors, and patients with dissecting, fusiform, and fungal types of IAs. (5) Patients with malignant tumors, infectious diseases, autoimmune disease, systemic inflammation, and recent use of related drugs that may affect serum levels of inflammation. (6) Patients who refused to participate, incomplete neuroimaging evaluation and incomplete clinical data. All participants provided informed consent by telephone or in writing, and all experiment protocols were approved by the Hospital Review Committee of Henan Provincial People’s Hospital. This study follows the principles of the Declaration of Helsinki.

### Data collection

We reviewed the electronic medical records of the medical system and collected relevant information of patients with small SIAs, including demographic baseline characteristics such as age, gender, hyperlipidemia, diabetes, hypertension, coronary heart disease, smoking, and drinking, aneurysm-related characteristics such as aneurysm location, number, and shape, as well as laboratory hematological parameters related indicators for the first time after admission such as blood routine examination, liver and kidney function, coagulation function, etc., and standardized the above clinical data. The serological test specimens of all patients were collected from the venous blood of the median cubital vein on the day of admission. All specimens were tested in a sterile environment in strict accordance with the kit instructions. The serological composite markers were expressed as follows: WPR = white blood cell count/platelet count*100, SII = neutrophil count*platelet count/lymphocyte count.

### Statistical analysis

The data of this study were statistically analyzed by SPSS statistical package 21.0 (IBM, USA) and plotted by Prism 5(GrafPad Software Inc. USA). First, Kolmogorov-Smirnov was used to conduct the normality test for all continuous variables, the continuous variables that met the normality test were represented by mean ± standard deviation $( \overline{x} \pm {\text{s}} )$, and the difference between groups was analyzed by the T-test of two independent samples, the continuous variables of skewed distribution were described by median and interquartile range, and Mann-Whitney U test was used to analyze the differences between groups, the categorical variables were expressed as quantities and percentages (n,%), and Fisher’s exact test or Pearson’s Chi-square test (χ^2^) compared the differences between groups, *p* < 0.05 was considered statistically significant between the two groups. The basic parameters of all patients with SIAs were analyzed by univariate and multivariate analysis, the correlation between serum inflammatory markers and unstable aneurysms was evaluated, the receiver operating characteristic (ROC) curve of independent risk factors and combined prediction and diagnosis of intracranial aneurysm instability was drawn, and the area under the curve (AUC), best cut-off point, sensitivity, and specificity were calculated. In addition, we performed an analysis of the differences in newly discovered inflammatory markers between unstable symptomatic unruptured aneurysms and ruptured aneurysms, plotted ROC curves, and compared the AUC.

## Results

### Baseline characteristics of SIAs instability

The population baseline and aneurysm-related characteristics of the patients are shown in Table 1. A total of 275 patients were included in the study, there were 187 patients with unstable aneurysms, including 76 patients (40.64%) over 60 years old and 119 females (63.64%). There were 88 patients with stable aneurysms, 32 patients (33.36%) over 60 years old, and 71 females (80.68%). Compared with patients with stable aneurysms, patients with unstable aneurysms had a higher proportion of males (*p* = 0.005) and a higher proportion of high-risk factors combined with hypertension (*p* = 0.02), hyperlipidemia (*p* = 0.006), history of ischemia (*p* = 0.025) and smoking (*p* = 0.004), usually occurs in the posterior circulation artery (*p* < 0.001), and the shape was mostly irregular (*p* < 0.001). Stable aneurysms were more likely to occur in the internal carotid artery and middle cerebral artery, and their morphology tended to be regular, with no statistical difference in age, coronary heart disease, diabetes, drinking history, family history, and number of aneurysms (*p* > 0.05).

**Table 1:** Baseline and aneurysm characteristics of all patients with small saccular intracranial aneurysms.

Variables	Total patients(*n* = 275)	Stable group (*n* = 88)	Unstable group (*n* = 187)	*p* Value
Gender				**0.005**
Female	190 (69.1%)	71 (80.68%)	119 (63.64%)	
Male	85 (30.9%)	17 (19.32%)	68 (36.36%)	
Age (years)				0.512
< 60	167 (60.73%)	56 (63.64%)	111 (59.36%)	
≥ 60	108 (39.27%)	32 (36.36%)	76 (40.64%)	
Hypertension	132 (48%)	33 (37.5%)	99 (52.94%)	**0.02**
Diabetes	32 (11.64%)	10 (11.36%)	22 (11.76%)	0.923
Heart disease	26 (9.45%)	7 (7.95%)	19 (10.16%)	0.662
Hyperlipidemia	47 (17.09%)	7 (7.95%)	40 (21.39%)	**0.006**
Previous stroke history	25 (9.09%)	3 (3.41%)	22 (11.76%)	**0.025**
Smoking	57 (20.73%)	9 (10.23%)	48 (25.67%)	**0.004**
Drinking	48 (17.45%)	12 (13.64%)	36 (19.25%)	0.308
Family history	9 (3.27%)	1 (1.14%)	8 (4.28%)	0.28
Aneurysm location				**< 0.001**
ICA	92 (33.45%)	46 (52.27%)	46 (24.59%)	
ACA	15 (5.45%)	1 (1.14%)	14 (7.49%)	
MCA	33 (12%)	13 (14.77%)	20 (10.69%)	
ACoA	45 (16.36%)	6 (6.82%)	39 (20.86%)	
PCoA	70 (25.45%)	18 (20.45%)	52 (27.81%)	
Posterior circulation artery	20 (7.27%)	4 (4.55%)	16 (8.56%)	
Number of aneurysms (multiple)	47 (17.09%)	16 (18.18%)	31 (16.58%)	0.734
Aneurysm shape				**< 0.001**
Regular	124 (45.09%)	59 (67.05%)	65 (34.76%)	
Irregular	151 (54.91%)	29 (32.95%)	122 (65.24%)	

***Stable group*** all unsymptomatic unruptured aneurysms were found accidentally, ***Unstable group*** symptomatic unruptured aneurysms and ruptured aneurysms, ***ICA*** internal carotid artery, ***ACA*** anterior cerebral artery, ***MCA*** middle cerebral artery, ***ACoA*** anterior communication artery, ***PCoA*** posterior communicating artery.

Significant values are in bold.

### Laboratory serum detection of SIAs instability

Serological test results of all patients are shown in Table 2. Univariate analysis showed white blood cells (*p* < 0.001), neutrophils (*p* < 0.001), lymphocytes (*p* < 0.001), homocysteine (*p* < 0.001), glucose (*p* < 0.001), D-dimer (*p* < 0.001), fibrinogen (*p* = 0.007), neutrophil to lymphocyte ratio (*p* < 0.001), platelet to lymphocyte ratio (*p* < 0.001), leukocyte to platelet ratio (*p* < 0.001), and systemic immune-inflammatory response index (*p* < 0.001) were closely related to small SIAs instability. There were no significant differences in other serum indexes such as monocyte, erythrocyte, hemoglobin concentration, mean platelet volume, total cholesterol, and triglyceride (*p* > 0.05).

**Table 2:** Laboratory serum detection of all patients with small saccular intracranial aneurysms.

Variables	Total patients (*n* = 275)	Stable group (*n* = 88)	Unstable group (*n* = 187)	*p* Value
WBC	6.41 (5.11,9.15)	5.81 (4.87,6.49)	7.3 (5.25,10.6)	**< 0.001**
Neutrophil	3.99 (2.91,7.56)	3.11 (2.69,3.71)	5.9 (3.14,9.97)	**< 0.001**
Lymphocyte	1.59 ± 0.67	2.09 ± 0.54	1.37 ± 0.6	**< 0.001**
Monocyte	0.38 (0.28,0.5)	0.37 (0.31,0.44)	0.38 (0.26,0.54)	0.71
RBC	4.35 ± 0.49	4.35 ± 0.45	4.35 ± 0.51	0.931
MCHC	330.27 ± 12.6	328.92 ± 9.6	330.91 ± 13.76	0.222
RDW-CV	12.5 (12.1,13.1)	12.4 (12.1,12.9)	12.6 (12.1,13.2)	0.131
PLT	228.38 ± 65.73	238.09 ± 58.49	223.81 ± 68.55	0.093
MPV	10.3 (9.7,11)	10.35 (9.7,10.98)	10.3 (9.7,11)	0.677
P-LCR	27.3 (23.0,32.9)	27.7 (22.2,32.35)	27.2 (23.7,33.3)	0.553
PDW	11.8 (10.7,13.4)	11.75 (10.17,13)	11.8 (10.8,13.5)	0.204
HCY	10.2 (8.7,13.2)	9.15 (7.3,10.5)	11.1 (9.2,15.1)	**< 0.001**
CHOL	4.29 ± 1.03	4.28 ± 1.06	4.3 ± 1.01	0.863
TG	1.26 (0.91,1.82)	1.28 (0.99,1.84)	1.26 (0.9,1.76)	0.469
GLU	5.58 (4.97,6.68)	5.22 (4.67,5.8)	5.8 (5.1,7.1)	**< 0.001**
D-Dimer	0.4 (0.22,0.86)	0.27 (0.19,0.35)	0.56 (0.27,1.35)	**< 0.001**
FBG	2.92 (2.55,3.42)	2.81 (2.46,3.22)	2.99 (2.58,3.52)	**0.007**
NLR	2.21 (1.52,7.12)	1.58 (1.19,1.88)	4.59 (1.98,11.22)	**< 0.001**
PLR	142.01 (110.18,201.9)	120.43 (90.42,141.34)	169.32 (120.22,257.48)	**< 0.001**
WPR	2.97 (2.17,4.26)	2.43 (1.94,3.03)	3.41 (2.35,5.21)	**< 0.001**
SII	542.32 (331.53,1555.27)	337.1 (261.66,498.01)	977.94 (417.58,2664.9)	**< 0.001**

***WBC*** white blood cell, ***RBC ***red blood cell, ***MCHC*** mean corpuscular hemoglobin concentration, ***RDW-CV*** red cell distribution width coefficient variation, ***PLT*** platelet count, ***MPV***: mean platelet volume, ***P-LCR*** platelet larger cell ratio, ***PDW*** platelet distribution width, ***HCY*** homocysteine, ***CHOL***cholesterol, ***TG*** triglyceride, ***GLU*** glucose, ***FBG*** fibrinogen, ***NLR*** neutrophil to lymphocyte ratio, ***PLR*** platelet to lymphocyte ratio, ***WPR*** white blood cell to platelet ratio, ***SII*** systemic immune-inflammatory index

Significant values are in bold.

### Multivariate analysis of SIAs instability

**Fig. 1:**
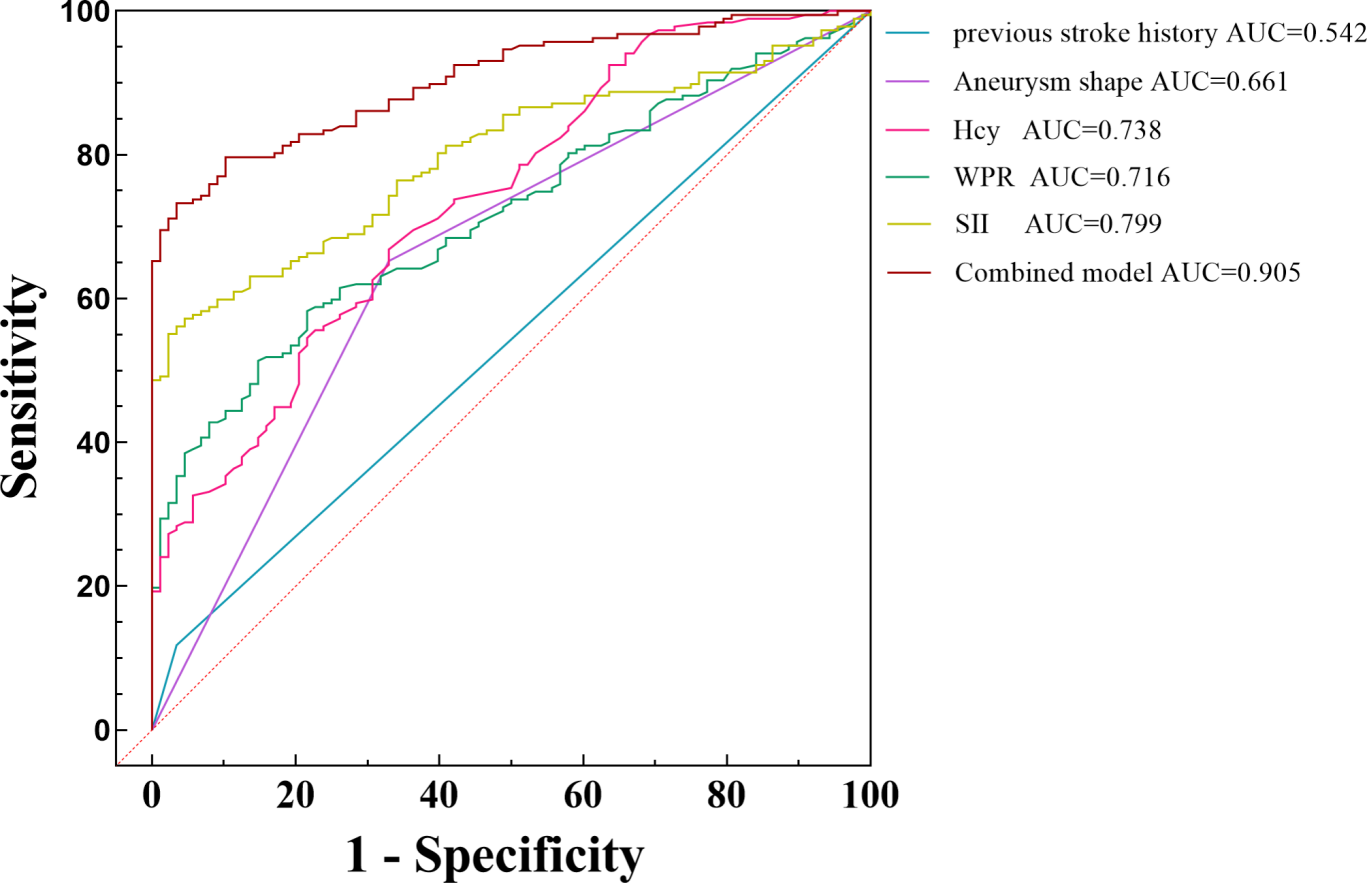
Previous stroke history, aneurysm shape, Hcy, WPR, SII, and combined prediction model were compared to predict the probability of secondary unstable progression of stable SIAs. Among them, the areas under curves of Hcy, WPR, SII and combined models were 0.738 (95%CI, 0.676–0.799, *p* < 0.001), 0.716 (95%CI, 0.656–0.776, *p* < 0.001), 0.799 (95%CI, 0.749–0.85, *p* < 0.001), 0.905 (95%CI, 0.871–0.939, *p* < 0.001).

Combined with baseline characteristics and serum test results, logistic regression analysis after adjusting for multiple confounding factors showed that irregular aneurysm morphology (OR = 3.295, *p* = 0.003), history of ischemia (OR = 5.71, *p* = 0.023), high homocysteine concentration (OR = 1.345, *p* < 0.001), WPR (OR = 2.468, *p* = 0.029), SII (OR = 1.006, *p* = 0.012) were independent risk factors for small SIAs instability. Table 3. The ROC curve of each independent risk factor for predicting small SIAs instability is shown in Fig. 1. The AUC of homocysteine predicting aneurysm instability was 0.738, the best critical value was 9.75, the sensitivity was 66.8%, and the specificity was 67%. The AUC of WPR was 0.716, the best critical value was 3.14, the sensitivity was 58.3%, and the specificity was 78.4%. The AUC of SII was 0.799, the best critical value was 837, the sensitivity was 55.1%, and the specificity was 97.7%. Table 4. The AUC of all high-risk factors combined to predict small SIAs instability was 0.905.

**Table 3:** Multivariate analysis of predictors related to the instability of small saccular intracranial aneurysms.

Variables	Beta	OR (95% CI)	*P* value
Previous stroke history	1.742	5.71 (1.277–25.523)	0.023
Aneurysm shape	1.192	3.295 (1.498–7.247)	0.003
HCY	0.296	1.345 (1.145–1.579)	< 0.001
WPR	0.904	2.468 (1.098–5.549)	0.029
SII	0.006	1.006 (1.0010–1.011)	0.012

*HCY* homocysteine, *WPR* white blood cell to platelet ratio, *SII* systemic immune-inflammatory index

**Table 4:** The predictive ability of SII, WPR and Hcy serum biomarkers for the unstable progression of small saccular intracranial aneurysms.

Variables	AUC	Optimal cut-off value	Sensitivity%	Specifcity%	Youden index
HCY	0.738	9.75	66.8	67	0.34
WPR	0.716	3.14	58.3	78.4	0.37
SII	0.799	837	55.1	97.7	0.53

HCY: homocysteine; WPR: White blood cell to platelet ratio; SII: systemic immune-inflammatory index

### Correlation analysis of serum SII, WPR, and hcy levels with instability and rupture of SIAs

**Fig. 2:**
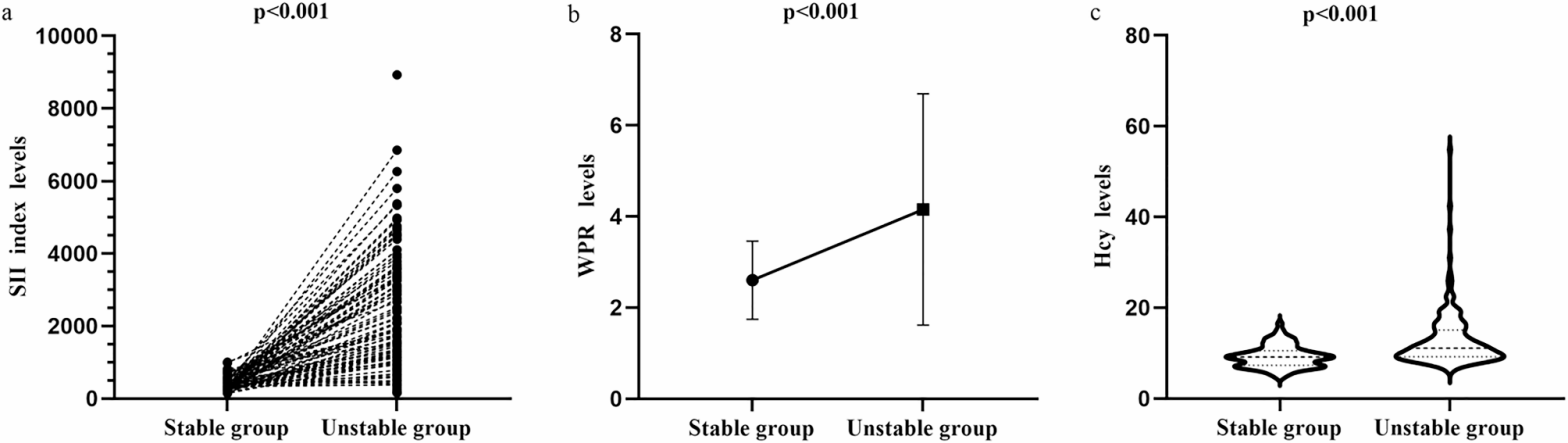
The difference of SII, WPR and Hcy levels between stable and unstable small SIAs patients in laboratory serological tests was compared. (**a**) The initial SII level was significantly different between the two groups (median 337.1 vs 977.94, *p* < 0.001). (**b**) The initial WPR level was significantly different between the two groups (median 2.43 vs 3.41, *p* < 0.001). (**c**) The initial Hcy level was significantly different between the two groups (median 9.15 vs 11.1, *p* < 0.001). Compared with patients with stable aneurysms, SII, WPR and Hcy in patients with unstable aneurysms were significantly higher.

**Fig. 3:**
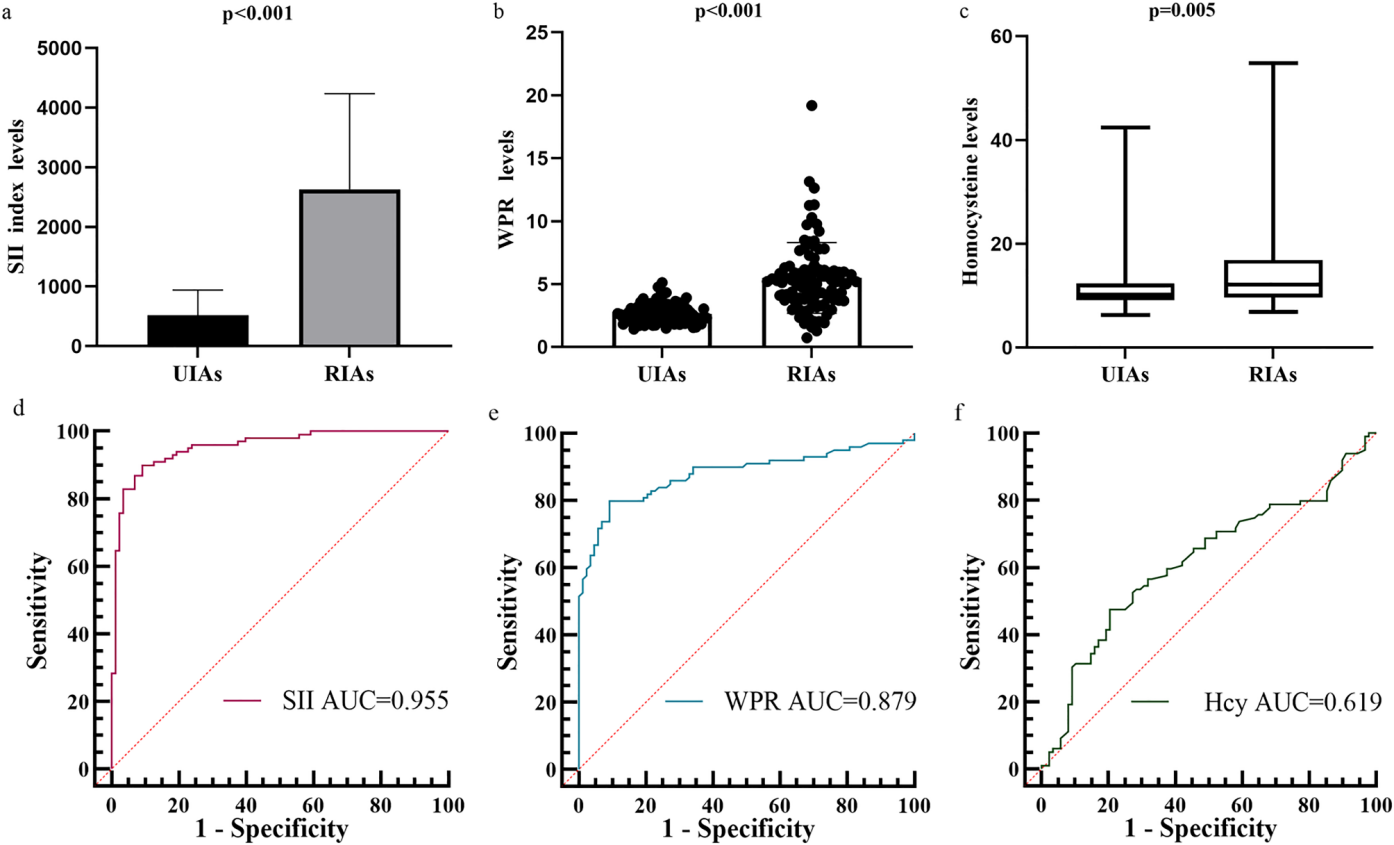
Differences in SII, WPR, and Hcy levels were compared between unruptured and ruptured patients in the unstable small SIAs group, and the area under the curve was plotted to predict the risk of aneurysm rupture. (**a**) The initial SII level was significantly different between the two groups(median 417.14 vs 2445.29, *p* < 0.001). (**b**) The initial WPR level was significantly different between the two groups (median 2.49 vs 5.19, *p* < 0.001). (**c**) The initial Hcy level was significantly different between the two groups (median 10.15 vs 12.1, *p* = 0.005). (**d**) The initial SII level predicted the probability of secondary rupture of unstable unruptured aneurysms was 0.955 (95%CI, 0.927–0.983). (**e**) The initial WPR level predicted the probability of secondary rupture of unstable unruptured aneurysms was 0.879 (95%CI, 0.828–0.931). (**f**) The initial Hcy level predicted the probability of secondary rupture of unstable unruptured aneurysms was 0.619 (95%CI, 0.538–0.7). Compared with patients with unruptured aneurysms, the SII, WPR and Hcy of patients with ruptured aneurysms were significantly higher. And it has a good predictive ability in the risk of secondary rupture in patients with unstable unruptured aneurysm types.

The differences of SII, WPR and Hcy levels in stable and unstable groups of small SIAs were analyzed by violin diagram and paired diagram respectively. The results showed that the levels of SII (337.1 vs 977.94, *p* < 0.001), WPR (2.43 vs 3.41, *p* < 0.001), and Hcy (9.15 vs 11.1, *p* < 0.001) in patients with unstable small SIAs were significantly higher than those in patients with stable asymptomatic small SIAs, and the difference was statistically significant (Fig. 2). The levels of SII, WPR and Hcy in different types of unstable small SIAs were analyzed by box diagram, the ROC curve was used to predict the risk of possible rupture of small SIAs. The results showed that the levels of SII (417.14 vs 2445.29, *p* < 0.001), WPR (2.49 vs 5.19, *p* < 0.001), Hcy (10.15 vs 12.1, *p* = 0.005) in patients with ruptured small SIAs were significantly higher than those in patients with symptomatic unruptured small SIAs. The AUC indicates that the above three indicators have good diagnostic ability in ruptured small SIAs (SII: AUC = 0.955, WPR: AUC = 0.879, Hcy: AUC = 0.619) (Fig. 3).

## Discussion

Our study found that in different aneurysm states of small SIAs, unstable patients showed higher levels of SII, WPR and Hcy than stable patients. In the meantime, in patients with unstable small SIAs, the levels of the above three inflammatory indicators in patients with rupture were also significantly higher than those in unruptured symptomatic patients. These results indicate that high levels of SII, WPR, and Hcy may be associated with the instability of small SIAs.

According to previous studies, there are many risk factors related to the instability of IAs, such as gender, hypertension, smoking, drinking, the size, number, and location of IAs, and even morphological characteristics derived from imaging such as flatness, density, elongation and fluctuation index of some IAs^26,27^. However, these factors can only partially explain the risk of IAs instability and rupture. Although the mechanism of IAs occurrence and progression is difficult to predict, it is known that inflammatory factors play an important role in vascular inflammation and the progress and outcome of IAs^28^.

Inflammation is a defensive response of the body’s vascular system to infection or tissue damage. When inflammation occurs, the body will produce more white blood cells in a feedback manner than in a normal state. As the main participant and central mediator of the inflammatory response, white blood cells can migrate through endothelial cells to the site of infection or inflammation under the attraction and activation of chemokines, and then continue to infiltrate into the site of inflammation. However, persistent white blood cell reaction will further aggravate the degree of tissue damage and may prolong the process of inflammation^29^. In addition, with the exudation of white blood cells outside the vascular wall, inflammatory cells such as neutrophils, monocytes, and endothelial cells are activated, releasing inflammatory mediators such as leukotrienes, proinflammatory cytokines (IL-1 and TNF-α), platelet-activating factors, and oxygen free radicals. They cooperate and antagonize each other to jointly regulate the inflammatory network^30^. Lymphocytes are important cellular components of the body’s immune system, which are widely distributed in the body and serve as the first line of defense against invasion of the defense system. During inflammation, immune function decreases and lymphocytes decrease significantly, which can eventually lead to systemic immunosuppression and immune-inflammatory imbalance. As a multifunctional cell, platelet plays an important role in hemostasis and can accelerate blood coagulation. At the same time, it can produce and release some bioactive mediators such as arachidonic acid metabolites and histamine, promote vascular permeability and leukocyte exudation, and have many dynamic functions of connecting coagulation and mediating immune inflammation^31,32^. Inflammation and coagulation in the body are stable and well-coordinated biological systems. Systemic inflammation can induce the activation of the coagulation system, and coagulation activation can reversely amplify the inflammatory effect, the two sides influence each other^33^. Systemic inflammation can affect the progression and prognosis of IAs diseases. Considering that the existing serum inflammatory markers are less studied in the progression of IAs disease, especially the most common small SIAs in clinical practice. Therefore, the purpose of our study is to explore the role of simple serological inflammation and inflammation-related composite indicators in the unstable progression of small SIAs, so as to promote the rapid identification and functional evaluation of high-risk patients with confirmed small SIAs.

SII and WPR are novel serum inflammatory composite markers proposed in recent years and have not attracted much attention in the progression of stable IAs. These two serum indicators combine white blood cells, neutrophils, platelets, and lymphocytes to a certain extent. As simple, economical, and easy to obtain indicators, they combine inflammation and coagulation factors and can reflect the body’s inflammatory state. Hu found that SII, as a comprehensive indicator of systemic inflammatory response, can reflect the balance between host inflammation and immune status in a relatively comprehensive way, and is closely related to poor prognosis and early recurrence after hepatocellular carcinoma^34^. In addition, SII has been shown in numerous studies to be a predictor of the progression and poor prognosis of a range of inflammatory diseases, including cardiovascular disease, inflammatory bowel disease, severe infections, and especially malignancy- related diseases^35–38^. A large retrospective study on aneurysmal subarachnoid hemorrhage by Yun et al. found that the SII index had a significant difference in the outcome of aSAH patients (815 × 10^9^/L vs. 1044 × 10^9^/L, *p* = 0.007), and was an independent risk factor for poor prognosis in aSAH patients, the best cut-off point is 960 × 10^9^/L (AUC:0.702, 95%CI:0.638–0.765, *p* < 0.001)^39^. Geraghty’s study also found that early SII index elevation after aSAH can independently predict delayed cerebral vasospasm, which is a serious adverse postoperative complication (AUC = 0.767, *p* < 0.001)^40^. In contrast, our study found for the first time the predictive ability of the SII index in the instability of small SIAs, including unruptured symptomatic SIAs and ruptured SIAs. As a potential biomarker of vascular inflammation, WPR has gradually attracted people’s attention in recent years, mainly in terms of cardiovascular and cerebrovascular risks and deterioration of liver and kidney function^41,42^, but there are relatively few studies on aneurysm diseases. Kim et al. found that high levels of WPR were associated with a higher risk of IAs rupture and a higher PHASES score(AUC,0.827, *p* < 0.001)^43^, although the sample size of this retrospective study is large enough, it includes all types of aneurysms and there are significant differences in the number of enrollments (1001 cases vs. 208 cases), considering that such a choice may cause certain deviations in the results, our study makes up for this deficiency to the greatest extent at the beginning of the design and strives to minimize bias and homogenize aneurysm types. In addition, our previous retrospective study found that high levels of WPR independently predicted DCI after aSAH, and the model showed good predictive power when combined with other platelet-related measures (combined model AUC = 0.875)^44^. Consistent with the purpose of the latest findings in this study, it provides new insights into the clinical treatment of IAs and helps to predict the development of the disease.

High levels of Hcy can indicate the body’s inflammatory state to a certain extent, but there is still a lack of understanding of the pathological mechanism of tissue damage caused by hyperhomocysteinemia. As a non-protein sulfur-containing amino acid formed after methionine demethylation in the human body, Hcy is an important intermediary factor in the metabolism of methionine and cysteine^45^. When the metabolic pathway is abnormally altered or blocked, dysregulation of the homocysteine-methionine cycle eventually leads to elevated plasma homocysteine levels^46^. Hyperhomocysteinemia in serum can promote the formation of oxygen free radicals and hydrogen peroxide, induce the up-regulation of cathepsin to promote the release of inflammatory cytokines such as IL-8 from endothelial cells, cause vascular endothelial cell injury and toxic effects, and then induce vascular inflammatory response^47^. In addition, it can enhance platelet activity and aggregation ability, leading to a range of artery-related diseases. Numerous previous studies have linked hyperhomocysteinemia to an increased risk of ischemic stroke in adults, cardiovascular disease, parkinson’s disease, and chronic kidney disease^48–51^. In the latest study, Peng et al. found for the first time that higher Hcy concentration can independently predict the compression symptoms associated with unruptured fusiform intracranial aneurysms (FIAs), which helps to deepen the understanding of the mechanism of FIAs^12^. In this study, we found that serum Hcy is involved in the progression of small SIAs and can be used as a predictive biomarker for unstable progression and rupture of small SIAs.

Although endovascular treatment has made breakthrough progress in IAs diseases, it is still challenging to monitor the progression of the disease. Considering that there are few studies on the stability of the most common small SIAs in clinical practice, our study predicts the unstable progression and rupture of small SIAs based on demographic baseline characteristics and clinically available laboratory examination indicators. The results showed that high levels of SII, WPR, and Hcy had a good predictive effect in predicting the unstable progression of aneurysms (SII: AUC = 0.799, WPR: AUC = 0.716, Hcy: AUC = 0.738). Compared with other scholars, we predicted the risk of aneurysm rupture by comparing the baseline differences in aneurysm patients with previous stroke history and aneurysm morphology. Our study found that the above risk factors were involved in the unstable progression of IAs disease, including but not limited to IAs rupture. Moreover, the combined diagnosis of the above five risk factors had better predictive ability for aneurysm instability (combined diagnosis: AUC = 0.905). Secondly, compared with unruptured symptomatic small SIAs, SII, WPR, and Hcy showed a higher tendency in ruptured small SIAs (*p* < 0.05), suggesting that SII, WPR, and Hcy have potential application value as simple diagnostic tools in predicting the unstable progression and rupture risk of small SIAs.

Even so, this study still has several limitations. First of all, this is a clinical retrospective study from a single center, some confounding factors in the data collection process may cause selection bias in the results, and this conclusion needs to be verified in future multi-center studies. Secondly, the inflammatory markers involved in this study were the results of the first blood collection at admission, we failed to record the dynamic changes of inflammatory markers because the inflammatory response may change over time.

## Conclusion

Our study found the predictive value of new inflammatory markers in patients with small SIAs. High serum levels of SII, WPR, and Hcy are associated with unstable symptoms and rupture of small SIAs, which provides an economical, reliable, easily accessible, and non-invasive method to evaluate the systemic inflammatory status, helps early identification and evaluation of patients at high risk of unstable small SIAs, and provides new insights into the clinical treatment strategies for such patients.

## Data Availability

The first author can provide unreserved raw data to support the conclusion of this paper under reasonable circumstances.
